# The facilitating effects of KRT80 on chemoresistance, lipogenesis, and invasion of esophageal cancer

**DOI:** 10.1080/15384047.2024.2302162

**Published:** 2024-01-19

**Authors:** Wen-Jing Yun, Jun Li, Nan-Chang Yin, Cong-Yu Zhang, Zheng-Guo Cui, Li Zhang, Hua-Chuan Zheng

**Affiliations:** a Department of Oncology, The Affiliated Hospital of Chengde Medical University, Chengde, China; b Department of Thoracic Surgery, Shandong Provincial Hospital, Jinan, China; c Department of Thoracic Surgery, The First Affiliated Hospital of Jinzhou Medical University, Jinzhou, China; d Cancer Center, The First Affiliated Hospital of Jinzhou Medical University, Jinzhou, China; e Department of Environmental Health, University of Fukui School of Medical Sciences, Fukui, Japan

**Keywords:** Esophageal cancer, KRT80, biological behaviors, prognosis, gene therapy

## Abstract

Keratin 80 (KRT80) is a filament protein that makes up one of the major structural fibers of epithelial cells, and involved in cell differentiation and epithelial barrier integrity. Here, KRT80 mRNA expression was found to be higher in esophageal cancer than normal epithelium by RT-PCR and bioinformatics analysis (*p* < .05), opposite to KRT80 methylation (*p* < .05). There was a negative relationship between promoter methylation and expression level of KRT80 gene in esophageal cancer (*p* < .05). KRT80 mRNA expression was positively correlated with the differentiation, infiltration of immune cells, and poor prognosis of esophageal cancer (*p* < .05). KRT80 mRNA expression was positively linked to no infiltration of immune cells, the short survival time of esophageal cancers (*p* < .05). The differential genes of KRT80 mRNA were involved in fat digestion and metabolism, peptidase inhibitor, and intermediate filament, desosome, keratinocyte differentiation, epidermis development, keratinization, ECM regulator, complement cascade, metabolism of vitamins and co-factor (*p* < .05). KRT-80-related genes were classified into endocytosis, cell adhesion molecule binding, cadherin binding, cell–cell junction, cell leading edge, epidermal cell differentiation and development, T cell differentiation and receptor complex, plasma membrane receptor complex, external side of plasma membrane, metabolism of amino acids and catabolism of small molecules, and so forth (*p* < .05). KRT80 knockdown suppressed anti-apoptosis, anti-pyroptosis, migration, invasion, chemoresistance, and lipogenesis in esophageal cancer cells (*p* < .05), while ACC1 and ACLY overexpression reversed the inhibitory effects of KRT80 on lipogenesis and chemoresistance. These findings indicated that up-regulated expression of KRT80 might be involved in esophageal carcinogenesis and subsequent progression, aggravate aggressive phenotypes, and induced chemoresistance by lipid droplet assembly and ACC1- and ACLY-mediated lipogenesis.

## Introduction

The incidence of esophageal cancer (EC) is increasing with the environmental and dietary changes. The most common types of esophageal cancer are adenocarcinoma and squamous cell carcinoma, which develop in different parts of the esophagus and are driven by different genetic changes. The squamous cell carcinoma is more predominant than adenocarcinoma. Its risk factors are composed of older age, male gender, smoking, alcohol use, polycyclic aromatic hydrocarbons (PAHs), gastroesophageal reflux disease (GERD), dysplasia, and teeth loss. Nonsteroidal anti-inflammatory drugs, vitamins, and vegetable, green tea, and fruit intake can prevent the esophageal carcinogenesis. Management of EC depends on patient fitness and tumor stage, endoscopic removal was used for early tumors, while chemotherapy, chemo-radiotherapy, surgical resection, or combinations of these were used for advanced tumors.^1–3^ Despite improvements in the management and treatment of EC patients, the general outcome remains very poor. Therefore, it was of the potential for therapeutic interventions to find out the biomarker and molecular target.

Extracellular matrix proteins, adhesion molecules, and cytoskeletal proteins form a dynamic network interacting with signaling molecules as an adaptive response to altered gravity. Keratins are intermediate filament cytoskeletal proteins of epithelial cells that are responsible for their structural integrity, and considered as representative markers for epithelial cells, and molecular markers for the diagnosis of basal cell carcinoma, oral squamous cell carcinoma, bladder cancer, breast cancer, hepatocellular carcinoma, cervical cancer and gastric adenocarcinoma. Keratins can be divided into two types: 28 acidic or type I (KRT9KRT40) and 26 basic or neural type II (KRT1-KRT8, KRT71-KRT86). Keratin 80 (KRT80) gene is located on chromosome 12q13 and encodes a 452-amino-acid protein with a calculated molecular mass of 50.5 kD and an isoelectric point of 5.47. Another smaller alterative variant encompasses only 422 amino acids and has a calculated molecular mass of 47.2 kDa and an isoelectric point of 5.08. KRT80 is a filament protein that makes up one of the major structural fibers of epithelial cells.^4–6^ This is also reflected in the non-α-helical KRT80 end domains that contains a relatively high number of proline and cysteine residues along with the complete absence of GGG or GGX repeats, typically found in the head and tail domains of most type II epithelial keratins. Ouyang et al.^7^ found that OTUB2 regulated KRT80 stability via deubiquitination through Lys-48 and Lys-63 and promoted tumor proliferation in gastric cancer by activating Akt pathway.

Langbein et al.^4^ have reported that KRT80 is structurally distinctly closer to type II hair keratins than to type II epithelial keratins. KRT80 expression is related to advanced tissue or cell differentiation. KRT80 containing intermediate filaments (IF) are located at the cell margins close to the desmosomal plaques, where they are tightly interlaced with the cytoplasmic IF bundles abutting there. In contrast, in cells entering terminal differentiation, KRT80 adopts the “conventional” cytoplasmic distribution. In evolutionary terms, KRT80 is one of the oldest keratins, demonstrable down to fish. In addition, KRT80 mRNA is subject to alternative splicing. Besides KRT80, they describe a smaller but fully functional splice variant KRT80.1, which arises only during mammalian evolution. Remarkably, unlike the widely expressed KRT80, the expression of KRT80.1 is restricted to soft and hard keratinizing epithelial structures of the hair follicle and the filiform tongue papilla. Rajagopalan et al.^8^ demonstrated that KRT80 overexpression was associated with skin hydration, including caspase 14 and filaggrin. Castellucci et al.^9^ revealed that KRT80 was lower in cutaneous leishmaniasis (CL) caused by Leishmania braziliensis than the control according to GWAS. Here, we for the first time clarified the clinicopathological significances and related molecular mechanisms of KRT80 expression in esophageal cancer.

## Materials and methods

### Cell culture and transfection

Esophageal squamous (KYSE-150) carcinoma cell lines come from the Cell Bank of the Chinese Academy of Sciences, Shanghai, China. They were maintained in RPMI 1640 medium supplemented with 10% fetal bovine serum (FBS), 100 units/mL penicillin, and 100 μg/mL streptomycin in a humidified atmosphere of 5% CO_2_ at 37°C. KRT80 siRNA (sc -96,163, Santa Cruz) was used to knockdown KRT80 in KYSE-150 cells using Lipofectamine 3000 (Thermo Fisher Scientific). The cells were treated with 5-Fluorouracil (5-FU, thymidylate synthetase inhibitor), or Taxol (a mitotic inhibitor), cycloheximide (CHX, a selective inhibitor of protein synthesis), Actinomycin D (a selective inhibitor of RNA synthesis) or MG132 (a proteasomal inhibitor).

### Proliferation assay

Cell Counting Kit-8 (CCK-8) was employed to determine the number of viable cells. In brief, 2.0 × 10^3^ cells/well were seeded on 96-well plate and allowed to adhere. At different time points, 10 μL of CCK-8 solution was added to each well of the plate and the plates were incubated for 3 h in the incubator, and then measured at 450 nm.

### Apoptosis assay by flow cytometry

Flow cytometry was performed with 7-amino-actinomycin (7-AAD) and phycoerythrin (PE)-labeled annexin V (BD Pharmingen, USA) to detect phosphatidylserine externalization as an endpoint indicator of early apoptosis as the protocol recommends.

### Wound healing assay

Cells were seeded at a density of 1.0 × 10^6^ cells/well in 6-well culture plates. After they had grown to confluence, the cell monolayer was scraped with a pipette tip to create a scratch, washed by PBS for three times and cultured in the FBS-free medium. Cells were photographed at 24 h and the scratch area was measured using Image software.

### Cell migration and invasion assays

For the migration assay, 2.5 × 10^5^ cells were resuspended in serum-free RPMI 1640, and seeded in the control-membrane insert on the top portion of the chamber (BD Bioscience). The lower compartment of the chamber contained 10% FBS as a chemo-attractant. After being incubated for 24 h, cells on the membrane were scrubbed, washed with PBS and fixed in 100% methanol and stained with Giemsa dye. For invasive assay, the procedures were the same as above, excluding the matrigel-coated insert (BD Bioscience).

### Nile red staining

We cultured cells on coverslips for 12 h, and incubated cells with Nile red (Invitrogen, 1:1000) for 15 min for 30 min. Slides were then fixed in 4% paraformaldehyde for 10 min. Finally, slides were stained with DAPI and mounted with SlowFade® Gold anti-fade reagent. Images were acquired and analyzed with the Image J and Icy software program.

### Co-immunoprecipitation (co-IP)

Eight μg of rabbit anti-ACC1 (Proteintech), rabbit anti-ACLY (Proteintech), or rabbit anti-ubiquitin (Proteintech) antibody was added to more than 1 mg of lysate protein and subjected to rotation at 4°C for more than 12 h. One hundred μL of agarose A/G beads were then added, and the mixture was rotated at 4°C for more than 12 h. To exclude nonspecific-binding proteins, the beads were centrifuged and washed with 1% NP40 lysis buffer three times. The pellet was mixed using 50 μL of 2× SDS sample buffer, and heated at 100°C for 18 min. The samples were used for the following western blot.

### Patients

Paraffin-embedded and frozen esophageal cancer and matched normal tissues were collected for the construction of tissue microarray and protein extraction from The First Affiliated Hospital of Jinzhou Medical University (China) between 2020 and 2021. Tissue microarrays of esophageal cancer and normal tissues were also purchased from Shanghai Outdo Biotech (Shanghai) and used for immunohistochemistry. None of the patients underwent chemotherapy, radiotherapy or adjuvant therapy before surgery. They all provided written consent for use of tumor tissue for clinical research and our University Hospital Ethical Committee approved the research protocol.

### Western blotting

RIPA lysis buffer was used to extract total proteins from fresh samples, which were then quantified using the BCA kit. Proteins of equivalent volume were separated by 10% SDS-PAGE and transferred to PVDF membranes. Nonspecific antigen sites were blocked by 5% skim milk for 1.5 h, and then incubated with rabbit anti-KRT80 (1:2000, Proteintech), mouse anti-Bcl-2 (1:500, Santa Cruz), mouse anti-Bax (1:500, Santa Cruz), rabbit anti-Akt (1:1000, Proteintech), rabbit anti- Caspase-1 (1:2000, Abcam), rabbit anti-E-cadherin (1:5000, Abcam), mouse anti-N-cadherin (1:2000, Abcam), rabbit anti-PI3K (1:1000, ABclonal), rabbit anti-PTEN (1:1000, HuaBio), rabbit anti-Twist1 (1:1000, Wanleibio), mouse anti-P53 (1:1000, CST), mouse anti- NF-κB (1:2000, Proteintech), mouse anti-Stat3 (1:2000, Proteintech), rabbit anti-CIDEA (1:1000, Proteintech), rabbit anti-CIDEB (1:2000, Proteintech), rabbit anti-CIDEC (1:2000, Proteintech), rabbit anti-ADRP (1:2000, Proteintech), rabbit anti-ACAT1 (1:1000, Huaan), rabbit anti-ACC1 (1:1000, proteintech), rabbit anti-ACLY (1:1000, proteintech), rabbit anti-IL-18 (1:1000, proteintech), rabbit anti-IL-1β (1:1000, Huaan), rabbit anti- Gasdermin D (1:1000, proteintech) rabbit anti-ubiqutin (1:1000, proteintech), mouse anti-COP1 (1:100, Santa Cruz), mouse anti-Nedd4 (1:100, Santa Cruz), or rabbit anti-GAPDH (1:2000, Hangzhou Goodhere) overnight at 4°C. After three washes, the membranes were treated with anti-rabbit or anti-mouse antibody with horseradish peroxidase (1:5000, CST, USA, #7074S) for 2 h. Protein bands were obtained with C300 (Azure Biosystems) by the Western Bright TM ECL western blotting detection kit (Advansta, USA, K-12045-D50), and analyzed by Image J software (v1.8.0).

### Tissue microarray (TMA)

Pathological specimens were fixed in 4% paraformaldehyde, dehydrated with alcohol, dealcoholized with xylene and embedded in paraffin. Paraffin blocks were sliced into 4 μm sections, and hematoxylin-and-eosin staining was used for histological analysis. Representative areas of adjacent normal tissues and solid tumors were identified by a microscope and corresponding tissue cores were punched out from paraffin blocks and transferred to pathological blocks, which were incised in 4 μm-thick.

### Immunohistochemistry (IHC)

The slides were deparaffinized and rehydrated three times, respectively, and antigen retrieval was completed in a microwave oven for 20 min. Three percent hydrogen peroxide (H_2_O_2_) and then 5% bovine serum albumin (BSA) were used to block endogenous peroxidase activity and nonspecific-binding sites for 30 min, respectively. Then, slides were incubated with rabbit anti-KRT80 (1:80, Abcam, USA, ab122605) for 3 h at room temperature. After rinsing with PBS for three times, the slides were incubated with polyclonal swine anti-rabbit antibody with HRP (1:200, DAKO, Japan, P0399) in room temperature for 2 h. diaminobenzidine (DAB) was used to visualize the specific-binding sites. After stained with hematoxylin, the slides were dehydrated, cleared, mounted, and visualized by a microscope (Nikon, Nikon Corporation, Japan). The evaluation of IHC was finished by the previous method.^10^

### Bioinformatics analysis

The expression and methylation of KRT80 gene was analyzed with the xiantao platform (https://www.xiantao.love/) and/or UALCAN database (http://ualcan.path. uab.edu). The prognostic significance of KRT80 was explored by Kaplan_Meier plotter (http://kmplot.com/). The differential genes were subjected to the construction of PPI network and selected for the important hub genes by cytoscape. These genes were subjected to GO+KEGG and GSEA analysis for the construction of signal pathways.

### Statistical analysis

Chi-square test, Spearman correlation analysis, and student *t*-test were used to compare the different rates and the means. Kaplan–Meier Curves were established with Log-rank test for univariate analysis and Cox’s hazard proportional model was used for multivariate analysis. SPSS 23.0 was used to conduct all statistical analyses. *p* < .05 was considered as statistically significant.

## Results

### The clinicopathological significances of KRT80 mRNA expression and methylation in esophageal cancer

**Figure 1.**
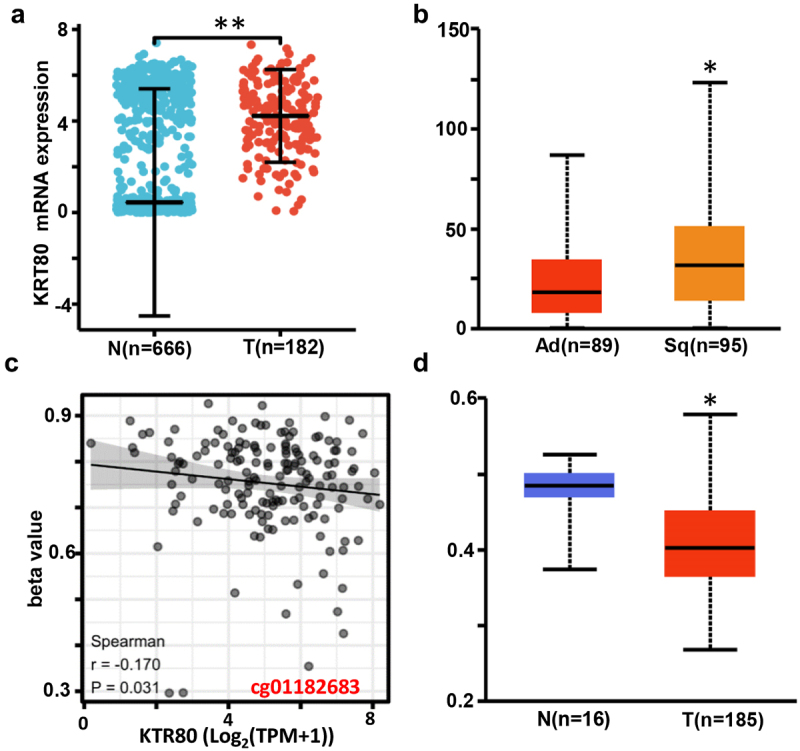
The clinicopathological and prognostic significances of KRT80 mRNA expression according to bioinformatics analysis. KRT80 mRNA expression was higher in esophageal cancer than normal tissue according to xiantao database (a), *p* < .05. It was compared with the histological subtypes of esophageal cancer by UALCAN (b) database. There was a negative relationship between KRT80 methylation and mRNA expression (c). KRT80 methylation level was lower in esophageal cancer than normal tissues by UALCAN (d), *p* < .05.

We found a higher expression of KRT80 mRNA in the cancer than normal epithelium of esophagus by xiantao database (Figure 1a, *p* < .05), and positively related to adenocarcinoma subtype of esophageal cancer by UALCAN database (Figure 1b, *p* < .05). There was a negative relationship between KRT80 mRNA and methylation (cg01182683) in esophageal cancer (Figure 1c, *p* < .05) in terms of xiantao database. The higher level of KRT80 methylation was observed in normal tissue than cancer (Figure 1d, *p* < .05).

ns, not significant; N, normal; T, tumor; Ad, adenocarcinoma; sq, squamous cell carcinoma

According to Kaplan–Meier plotter, KRT80 mRNA expression was negatively correlated with a long overall survival time of the cancer patients with stage 2, low mutation burden, decreased mesenchymal stem and natural killer cells (Supplementary Figure S1, *p* < .05), but the converse results were seen in those patients with stage 3 and high mutation burden (Supplementary Figure S1, *p* < .05). It was inversely linked to the relapse-free survival of the white, and B-cell-enriched cancer patients, while the converse was true for the cancer patients with low mutation burden, decreased B-cells, basophils and CD4+ T cells (Supplementary Figure S1, *p* < .05).

### The related genes and signal pathways of KRT80 in esophageal cancer

**Figure 2.**
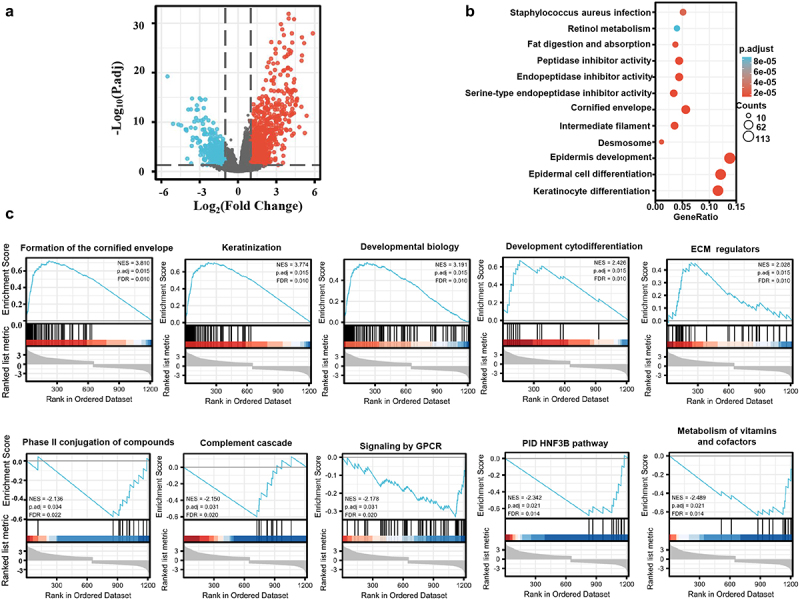
The differential genes and related signal pathways between low and high KRT80 expression in esophageal cancer. The volcano map of the differential genes was shown between low and high KRT80 expression in esophageal cancer (a). These genes were subjected to the signal pathway analysis using KEGG (b) and GSEA(c).

In xiantao platform, we found out the differential genes between low and high-expression groups of KRT80 mRNA in esophageal cancer and build up the volcano map as Figure 2a shows. KEGG analysis showed that the top-signal pathway included fat digestion and metabolism, peptidase inhibitor, and intermediate filament, desmosome, keratinocyte differentiation, epidermal cell differentiation, epidermis development, and so on (Figure 2b, *p* < .05). GSEA analysis showed that the top-signal pathways were composed of keratinization, ECM regulator, complement cascade, metabolism of vitamins and co-factor, and so forth (Figure 2c, *p* < .05). In addition, the STRING was used to identify the PPI pairs (Supplementary Figure S2a) and the cytoscape to find out the top 10 nodes ranked by degree (Supplementary Figure S2b). According to xiantao database, PPL, PI3, LCE3E, and TGM1 were more expressed in esophageal cancer than normal tissues (Supplementary Figure S2c, *p* < .05).

**Figure 3.**
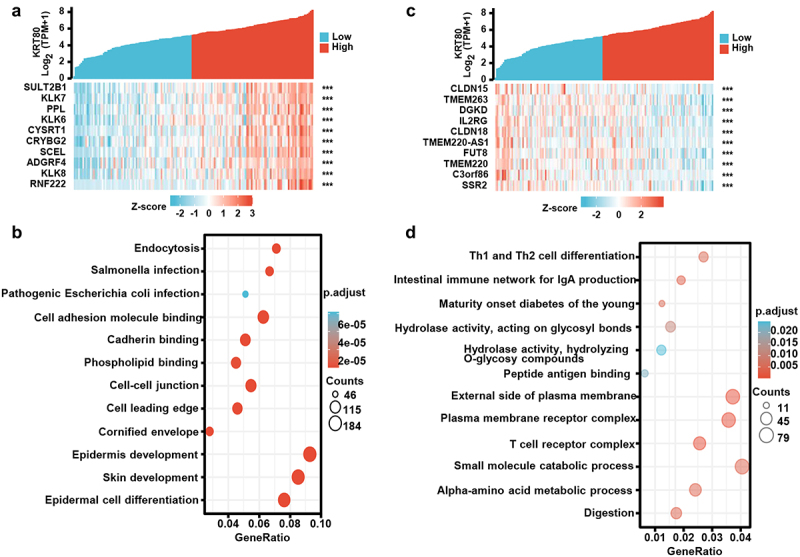
The KRT80-related genes and signal pathways in esophageal cancer. The positively related genes of KRT80 were screened (a), and were classified into the signal pathway using xiantao database (b). The negatively related genes of KRT80 were screened (c), and were classified into the signal pathway using xiantao database (d).

According to xiantao database, the positively correlated genes of KRT80 in esophageal cancer were shown in Figure 3a (*p* < .05), and involved in endocytosis, cell adhesion molecule binding, cadherin binding, cell–cell junction, cell leading ege, epidermal cell differentiation and development, and so forth (Figure 3b). The negatively correlated genes of KRT80 in esophageal cancer were shown in Figure 3c (*p* < .05), and involved in T cell differentiation, T cell receptor complex, immune network for IgA production, plasma membrane receptor complex, external side of plasma membrane, metabolism of amino acids and catabolism of small molecules, (Figure 3d). The positively correlated genes (SLUT2B1, PPL, KLK6, CRYBG2, SCEL, ADGRF4 and KLK8) were more frequently expressed in esophageal cancer than normal tissue (Supplementary Figure S3a, *p* < .05). Among negatively correlated genes, a higher expression of CLDNI5 and IL2RG, and was seen in esophageal cancer than normal tissue (Supplementary Figure S3b, *p* < .05), but the converse was true for DGKD, CLDNI8, TMEM220-AS1, TMEM220, C3orf86, and SSR2 (Supplementary Figure S3b, *p* < .0.05).

### The clinicopathological significances of KRT80 expression and methylation in esophageal cancer

**Figure 4.**
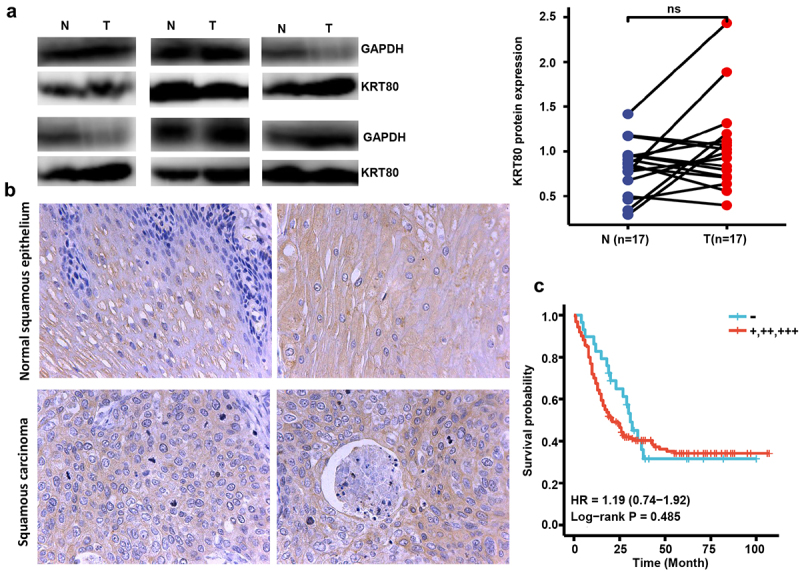
The clinicopathological significance of KRT80 protein expression in esophageal cancer. Western blot was used to detect KRT80 protein level in esophageal cancer (a). Densimetric analysis showed no difference in KRT80 expression between esophageal cancer and normal tissues (A, *p* > .05). Immunohistochemically, KRT80 protein was positively expressed in esophageal squamous epithelial and cancer cells (b). Kaplan–Meier curves and log-rank test were used to clarify the prognostic significance of KRT80 protein expression (c).

According to densitometric analysis of Western blot, no difference in KRT80 expression was found between esophageal cancer and matched normal tissues (Figure 4a, *p* > .05). Immunohistochemically, KRT80 protein was positively expressed in esophageal squamous epithelial cells and esophageal cancer cells (Figure 4b). As summarized in Table 1, the positive rates of KRT80 expression were 85.4% (252/295) and 87.2% (285/327) in esophageal normal tissues and cancers with no statistical significance (*p* > .05). Considering the frequency and density, KRT80 expression was stronger in young than elder cancer patients (*p* < .05), but not correlated with sex, histological grade, T stage, N stage or AJCC staging of esophageal cancers (Table 2, *p* > .05). Univariate analysis showed that sex, positive lymph node, T stage, N stage, AJCC stage and KRT80 were positively correlated with the unfavorable overall survival of the esophageal cancer patients (Figure 4c and Table 3, *p* < .05). Multivariate analysis showed that AJCC staging was an independent factor for the esophageal cancer patients (Table 3, *p* < .05).

N, normal; T, tumor; ns, not significant; HR, hazard ratio.

**Table 1.** KRT80 expression during esophageal carcinogenesis.

Groups	n	KRT80 expression				
		-	+	++	+++	PR (%)
Normal tissue	295	43	137	85	30	85.4
Esophageal cancer	327	42	144	102	39	87.2

PR, positive rate.

**Table 2.** The relationship between KRT80 protein expression and clinicopathological characteristics of esophageal cancer by immunohistochemistry.

Clinicopathological features	n	KRT80 expression				PR(%)	ρ	p value
		-	+	++	+++			
Sex							0.043	0.440
female	67	18	25	20	4	73.1		
male	142	33	119	82	35	76.8		
Age (years)							−0.163	**0.003**
＜65	183	18	75	61	29	90.2		
≥65	142	24	67	41	10	83.1		
T stage							0.019	0.729
T1	21	3	8	5	5	85.7		
T2	51	5	26	15	5	90.2		
T3	163	31	101	27	4	81.0		
T4	13	0	6	5	2	100.0		
N stage							0.037	0.506
N0	145	16	68	48	13	90.0		
N1	95	15	37	32	11	84.2		
N2	66	9	32	14	11	86.4		
N3	10	0	6	7	3	100.0		
Clinical stage							0.024	0.676
I	26	4	12	6	4	84.6		
II	122	10	58	43	11	91.8		
III	162	23	67	52	20	85.8		
IV	7	0	3	1	3	100.0		
Histological grade							−0.049	0.438
I	70	6	35	27	2	91.4		
II-III	188	31	89	47	21	83.5		

PR, positive rate.

**Table 3.** The survival analysis of the esophageal cancer patients by immunohistochemistry.

Variables	Univariate analysis			Multivariate analysis		
	β	HR (95% CI)	p-value	β	HR (95% CI)	p-value
Sex (male vs female)	0.598	1.819 (1.245–2.656)	**0.002**	0.623	1.864 (1.074–3.235)	**0.027**
Age (≥65 vs <65 years)	−0.17	0.874 (0.642–1.117)	0.24	−0.02	0.980 (0.653–1.471)	0.992
Positive lymph (≥2 vs < 2)	0.629	1.875 (1.411–2.491)	**<0.001**	−0.12	0.885 (0.467–1.678)	0.709
T stage (T1–2 vs. T3–4)	0.715	2.044 (1.339–3.120)	**0.001**	0.155	1.167 (0.655–2.081)	0.600
N stage (N0–1 vs N2–3)	0.807	2.241 (1.643–3.057)	**<0.001**	0.28	1.323 (0.714–2.452)	0.374
Clinical stage (I-II vs III-IV)	0.784	2.190 (1.635–2.934)	**<0.001**	0.757	2.131 (1.204–3.772)	**0.009**
Histological grade (I-II vs III)	0.018	1.019 (0.738–1.406)	0.911	0.209	1.232 (0.791–1.920)	0.356
KRT80 expression (- vs +~+++)	0.177	1.193 (0.722–1.974)	0.491	0.218	1.243 (0.711–2.175)	0.446

HR, hazard ratio; CI, confidence interval.

### The effects of KRT80 expression on the phenotypes of esophageal cancer cells

**Figure 5.**
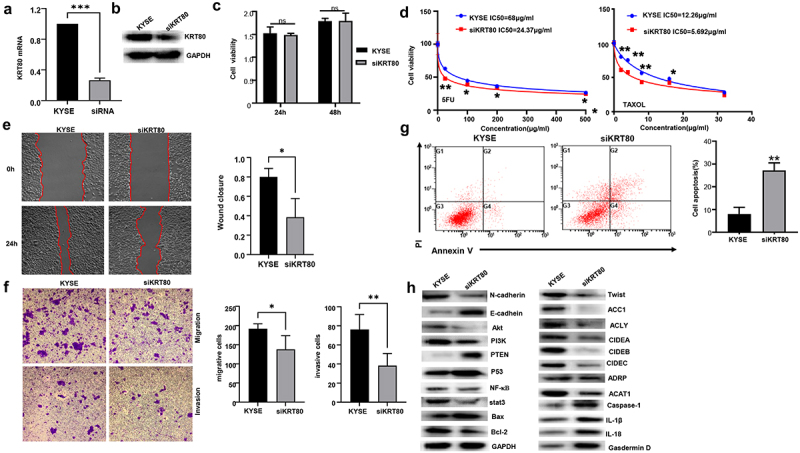
The effects of KRT80 knockdown on the aggressive phenotypes and phenotype-related protein of esophageal cancer cells. After transfection of siKRT80, KRT80 expression became weaker than the control in KYSE-150 cells by quantitative RT-PCR (a) and Western blot (b). The transfectants were subjected to the function assays of proliferation, chemosensitivity, migration and invasion, apoptosis by CCK-8 (c and d), wound healing (e) and transwell chamber (f), annexin V/7-AAD staining (g) respectively. The phenotype’s proteins were screened by Western blot (H).

After being transfected with siKRT80, KYSE-150 had a hypoexpressed KRT80 mRNA or protein expression by real-time RT-PCR (Figure 5a) or western blot (Figure 5b). There was no difference in growth rate between siKRT80 transfectants and control cells (Figure 5c, *p* > .05). KRT80 silencing caused the chemosensitivity to 5-FU and Taxol (Figure 5d). Compared with control cells, KRT80 under-expression decreased migration and invasion capacities of KYSE-150 cells by wound healing (Figure 5e, *p* < .05) and transwell assays (Figure 5f, *p* < .05). There was a higher apoptosis in KYSE-150 cells after siKRT80 transfection (Figure 5g, *p* < .05). As indicated in Figure 5h, KRT80 knockdown decreased the levels of expression of Akt, PI3K, NF-κB, stat3, Bcl-2, N-cadherin, Twist, ACC1, ACLY, CIDEC, CIDEB, CIDEA, ADRP, and ACAT1, but increased the levels of expression of PTEN, p53, Bax, Caspase-1, Gasdermin D, IL-18, IL-1β and E-cadherin in KYSE-150 cells.

KYSE, KYSE-150 *, *p* < .05; **, *p* < .01; ***, *p* < .001.

**Figure 6.**
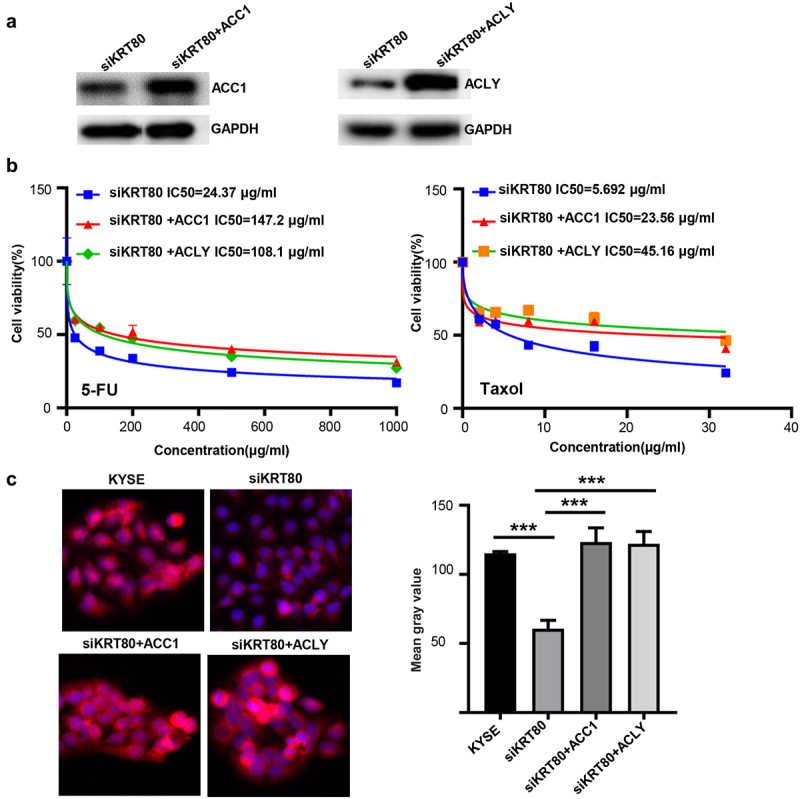
The effects of ACC1 and ACLY on the KRT80-mediated chemoresistance and lipogenesis. Either ACC1 and ACLY was overexpressed in siKRT80 transfectants of KYSE-150 cells, evidenced by Western blot (a). After the treatment of 5-FU and Taxol, KYSE-150 cells and transfectants were subjected to CCK-8 (b) and nile red staining (c) respectively.

KYSE, KYSE-150; ***, *p* < .001.

To verify the effects of ACC1 and ACLY on the KRT80-mediated chemoresistance and lipogenesis, we overexpressed ACC1 and ACLY, evidenced by Western blot (Figure 6a). Either ACC1 or ACLY increased the chemoresistance against 5-FU and Taxol and lipid droplet formation in siKRT80 transfectants of KYSE-150 cells by CCK-8 (Figure 6b, *p* < .05) and Nile red staining (Figure 6c, *p* < .05) respectively.

### The effects of KRT80 on the stability of ACC1 and ACLY mRNA or protein in esophageal cancer cells

**Figure 7.**
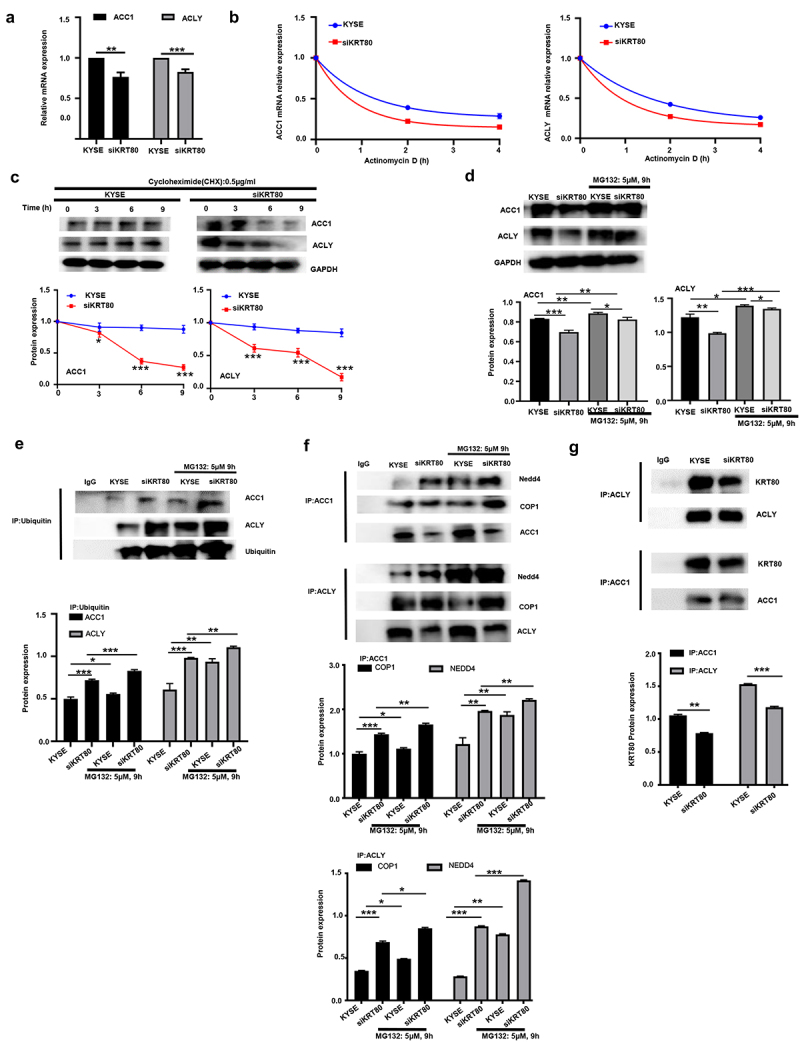
The effects of KRT80 on the stability of ACC1 and ACLY mRNA or protein. Either ACC1 or ACLY mRNA expression was hypoexpressed in siKRT80 transfectants of KYSE-150 cells, evidenced by real-time PCR (a). After the treatment of actinomycin D, we found that the stability of ACC1 and ACLY mRNA was reduced in siKRT80 transfectants (b). It was same for the stability of their encoding products, evidenced by Western bolt after the exposure to cycloheximide (CHX) (c). MG132 (a proteasomal inhibitor) treatment weakened the down-regulation of ACC1 and ACLY proteins in siKRT80 transfectants, compared with the control (d). Co-IP showed that KRT80 knockdown increased the ubiquitination of ACC1 and ACLY proteins (e), which might bind to ubiquitinases (COP1 and Nedd4, f and KRT80 protein (g).

Either ACC1 or ACLY mRNA expression was weakened in siKRT80 transfectants of KYSE-150 cells, evidenced by real-time PCR (Figure 7a, *p* < .05). After the treatment of Actinomycin D, we found that the stability of ACC1 and ACLY mRNA was alleviated in siKRT80 transfectants (Figure 7b, *p* < .05). After the exposure to cycloheximide, the stability of their encoding proteins was decreased, evidenced by Western bolt (Figure 7c, *p* < .05). MG132 treatment weakened the down-regulation of ACC1 and ACLY proteins in siKRT80 transfectants, compared with the control (Figure 7d, *p* < .05). Co-IP showed that KRT80 knockdown increased the ubiquitination of ACC1 and ACLY proteins (Figure 7e, *p* < .05), which might interact with such ubiquitinases as COP1 and Nedd4 (Figure 7f, *p* < .05) and KRT80 protein (Figure 7g, *p* < .05).

KYSE, KYSE-150; *,*p* < .05; **,*p* < .01; ***,*p* < .001.

## Discussion

As an adaptive reaction to changes in gravity, extracellular matrix proteins, adhesion molecules, and cytoskeletal proteins create a dynamic network that interacts with signaling molecules. KRT80 is a filamentous protein, one of the major structural fibers that make up epithelial cells, and is significantly closer in structure to type II hair keratin than to type II epithelial keratin. Nonetheless, it is to be found in almost all types of epithelial cells, interacts with intermediate filament bundles close to the desmosomal plaques, and involved in cellular differentiation.^4^ Although Castellucci et al.^9^ found that KRT80 mRNA expression was lower in cutaneous leishmaniasis than the control by GWAS, it was reported to up-regulate in ovarian, colorectal and gastric cancers at either mRNA or protein level.^6,7,10–12^ Here, we noticed that KRT80 mRNA expression was higher in esophageal cancer by bioinformatics analysis, indicating that up-regulated KRT80 expression might be involved in esophageal carcinogenesis. In agreement with the report in gastric cancer,^10^ a negative relationship between promoter methylation and the expression level of the KRT80 gene was found in esophageal cancer and KRT80 hypomethylation was observed in esophageal cancer, suggesting that KRT80 hypomethylation might be responsible for its up-regulation, which should be investigated in the future.

KRT80 expression was shown to be substantially related with increased lymph node and distant metastasis, as well as a higher pathological stage of colorectal cancer.^5,11^ KRT80 expression was significantly associated with lower disease-free survival, and overall survival in colorectal cancer patients as an independent prognostic indicator.^5^ Sanada et al.^13^ found that KRT80 was a prognostic factor for the lung adenocarcinoma patients. Liu et al.^12^ found that the expression levels of KRT80 were related to survival and prognosis as an independent prognostic factor in patients with ovarian cancer. Here, we found that KRT80 mRNA expression was negatively correlated with a long overall survival time of the cancer patients with stage 2, low mutation burden, decreased mesenchymal stem and natural killer cells, but the converse results were seen in those patients with stage 3 and high mutation burden. It was inversely linked to the relapse-free survival of B-cell-enriched cancer patients, while the converse was true for the cancer patients with low mutation burden, decreased B-cells, basophils and CD4+ T cells. The differential prognostic significance of KRT80 mRNA expression might be attributable the difference in the treatment approach and efficacy of the cancer patients with different stages, and different infiltration of immune cells.

Furthermore, KRT80 knockdown was reported to inhibit proliferation, anti-apoptosis, anti-pyroptosis, migration, invasion, and epithelial- mesenchymal transition (EMT) in gastric cancer cells.^10^ Tong et al.^14^ found that depletion of KRT80 restrained proliferation, migration, invasion, and EMT of lung cancer cells by affecting the TGF-β/SMAD pathway. Liu et al.^12^ found that miR-206/ETS1-mediated KRT80 overexpression promoted the proliferation, the transition from G_1_ phase to S phase, invasion, migration and EMT of ovarian cancer cells by MEK/ERK pathway. KRT80 expression promoted proliferation, migration, invasion and EMT of colorectal cancer cells by interacting with PRKDC via Akt pathway.^5^ Zhao et al.^15^ demonstrated that NSCAT1 competed with miR-1245 to suppress the inhibitory effects of miR-1245 on the translation of KRT80 in head and neck squamous cancer cells. KRT80 knockdown reduced the viability and of colorectal cells. KRT80 could interact with protein kinase, DNA-activated, catalytic polypeptide in colorectal cancer cells. KRT80-related genes were shown to be highly expressed in the cell cycle, DNA replication, immune system, protein, and RNA metabolism, signal transduction, and other cellular processes.^5,6^ CircPIP5K1A activated KRT80 to promote proliferation, invasion, migration, and EMT of gastric cancer cells,^16^ while TCONS_00049140 inactivated KRT80 and increased proliferation and melanin production of mouse melanocytes.^17^ Perone et al.^18^ demonstrated that SREBP1 drove Keratin-80-dependent cytoskeletal rearrangements and invasive behavior in endocrine-resistant ERα breast cancer. Rajagopalan et al.^8^ observed that KRT80 maintained epithelial barrier integrity in primary skin keratinocytes chronically exposed to cigarette smoke condensate. Our previous study showed that KRT80-related signal pathways were composed of ligand–receptor interaction, estrogen signal pathway, peptidase, filament and cytoskeleton, keratinocyte differentiation, vitamin D receptor, muscle contraction, B cell-mediated immune, cell adhesion and junction, and skin and epidermis development.^10^ Here, the KRT80-related genes were mostly involved in fat digestion and metabolism, peptidase inhibitor, and intermediate filament, desmosome, keratinocyte differentiation, epidermis development, extracellular matrix regulator, complement cascade, metabolism of vitamins, co-factor and amino acids, endocytosis, cell adhesion molecule binding, cell–cell junction, cell leading edge, plasma membrane receptor complex, and external side of plasma membrane, which account for the promoting effects of KRT80 on anti-apoptosis, migration, and invasion of esophageal cancer cells.

Opposite to p53, PI3K/Akt/NF-κB pathway is one of the most frequently over-activated intracellular pathways and involved in anti-apoptosis in various cancers, while PTEN inhibits PI3K/Akt signaling pathway.^19,20^ In apoptosis, Bcl-2 can interact with Bax on the mitochondrial membrane to suppress Bax-mediated opening of the mitochondrial voltage-dependent anion channel for apoptosis.^21^ In esophageal cancer cells, KRT80 silencing ameliorated proliferation and induced the apoptosis by inactivating PI3K/Akt/NF-κB, up-regulating PTEN and p53 or decreasing Bcl-2/Bax. Reportedly, pyroptosis is a recently discovered form of inflammatory programmed necrosis characterized by Caspase-1 -mediated cell death and its signal proteins are also composed of Gasdermin D, IL-18 and IL-1β.^22^ Twist is found to promote EMT with E-cadherin overexpression and N-cadherin under-expression.^23^ Therefore, we believed that KRT80 knockdown promoted the pyroptosis, and suppressed EMT of esophageal cancer cells by increasing slug and snail. KRT80 silencing reduced the expression of MMP-9, which account for the promoting effects of KRT80 on the invasion and metastasis of esophageal cancer cells.

KYSE-150 cells developed chemosensitivity to 5-FU and TAXOL and weakened the lipid droplet formation as a result of KRT80 knockdown. Reportedly, chemoresistance of colorectal cancer cells was produced by LPCAT2-mediated lipid droplet formation,^24^ which was also aided by prothymosin α,^25^ and metastasis-associated in colon cancer 1^26^ through SREBP-1- and FASN-mediated and lipogenesis respectively. A crucial enzyme for de novo fatty acid synthesis is either ACC1 or ACLY, which is closely linked to chemoresistance.^27^ In the liver and peritoneal tissues, lipid droplet assembly is mediated by ACAT1, ADRP and CIDEs.^28–30^ KRT80-mediated lipid droplet formation might be closely linked to the expression of ADRP, CIDEA, CIDEB, and CIDEC. KRT80-induced lipogenesis might be remarkably associated with the expression ACC1 and ACLY. Moreover, KRT80-mediated lipogenesis might account for the KRT80-induced chemoresistance against 5-FU and DDP because ACC1 and ACLY overexpression might reverse the inhibitory effects of KRT80 silencing on lipid droplet formation and chemoresistance. In combination of these discoveries, we hypothesized that KRT80 may have a role in chemoresistance by both de novo lipogenesis and lipid droplet assembly. Finally, KRT80 might stabilize the stability of ACC1 and ACLY mRNA, and their encoding proteins by inhibiting the proteasomal degradation possibly via their weakened interaction with the KRT80, COP1, and Nedd4.

In summary, KRT80 is believed to be involved in the pathogenesis and subsequent progression of esophageal cancer by promoting anti-apoptosis, anti-pyroptosis, migration, invasion, and EMT of esophageal cancer cells. KRT80 might induce the chemoresistance by lipid droplet assembly and ACC1- and ACLY-mediated lipogenesis in esophageal cancer cells.

## Supplementary Material

Supplemental Material

## Data Availability

The datasets used and/or analyzed are available from the corresponding author on reasonable request.
