# Supplementary figures and images for: The facilitating effects of KRT80 on chemoresistance, lipogenesis, and invasion of esophageal cancer
Source: Cancer Biol Ther. 2024 Jan 19;25(1):2302162. doi: 10.1080/15384047.2024.2302162 (PMC10802210; doi:10.1080/15384047.2024.2302162)

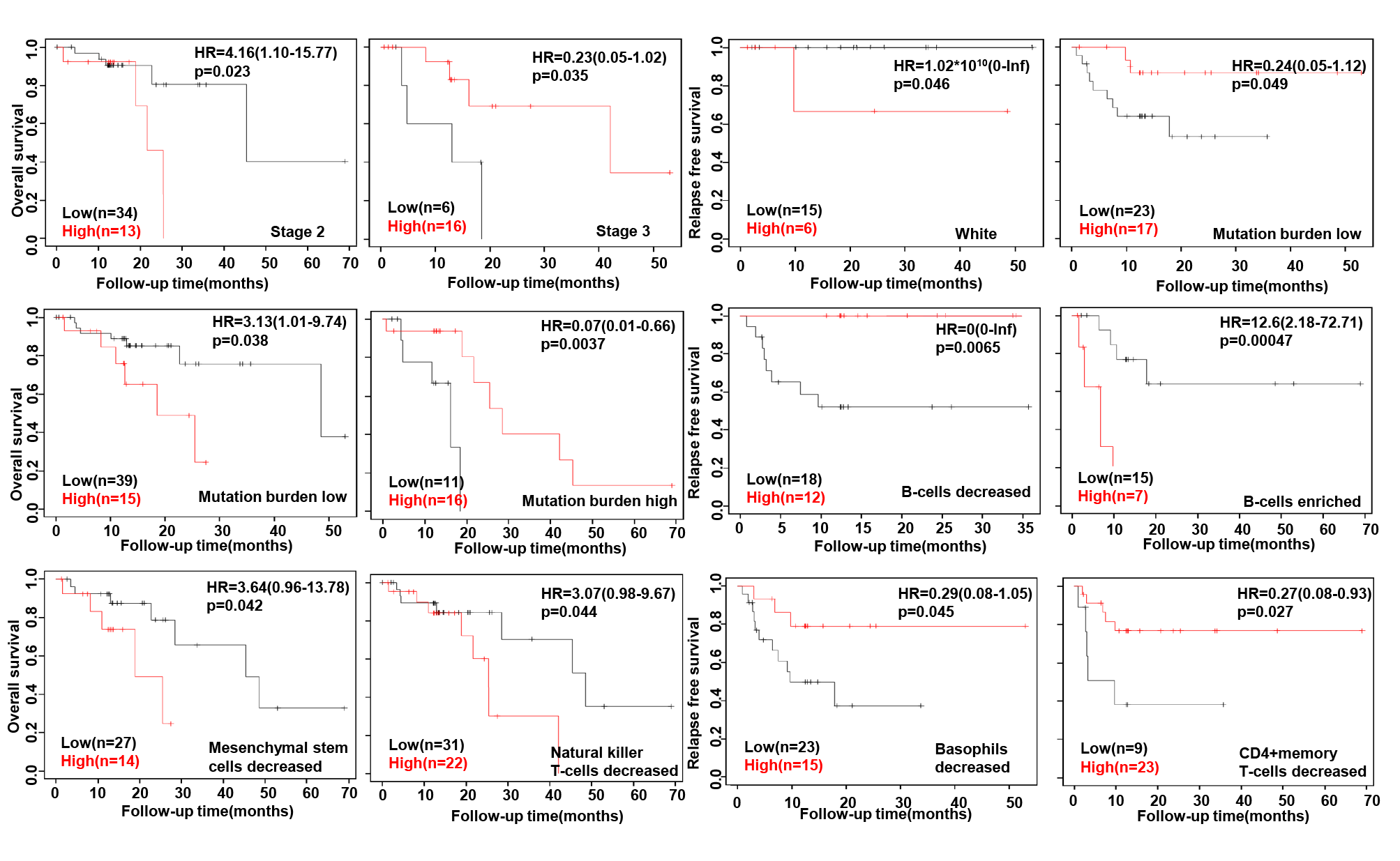

Supplement: Supplemental Material [file KCBT_A_2302162_SM7056.zip › Figure S1.tif]

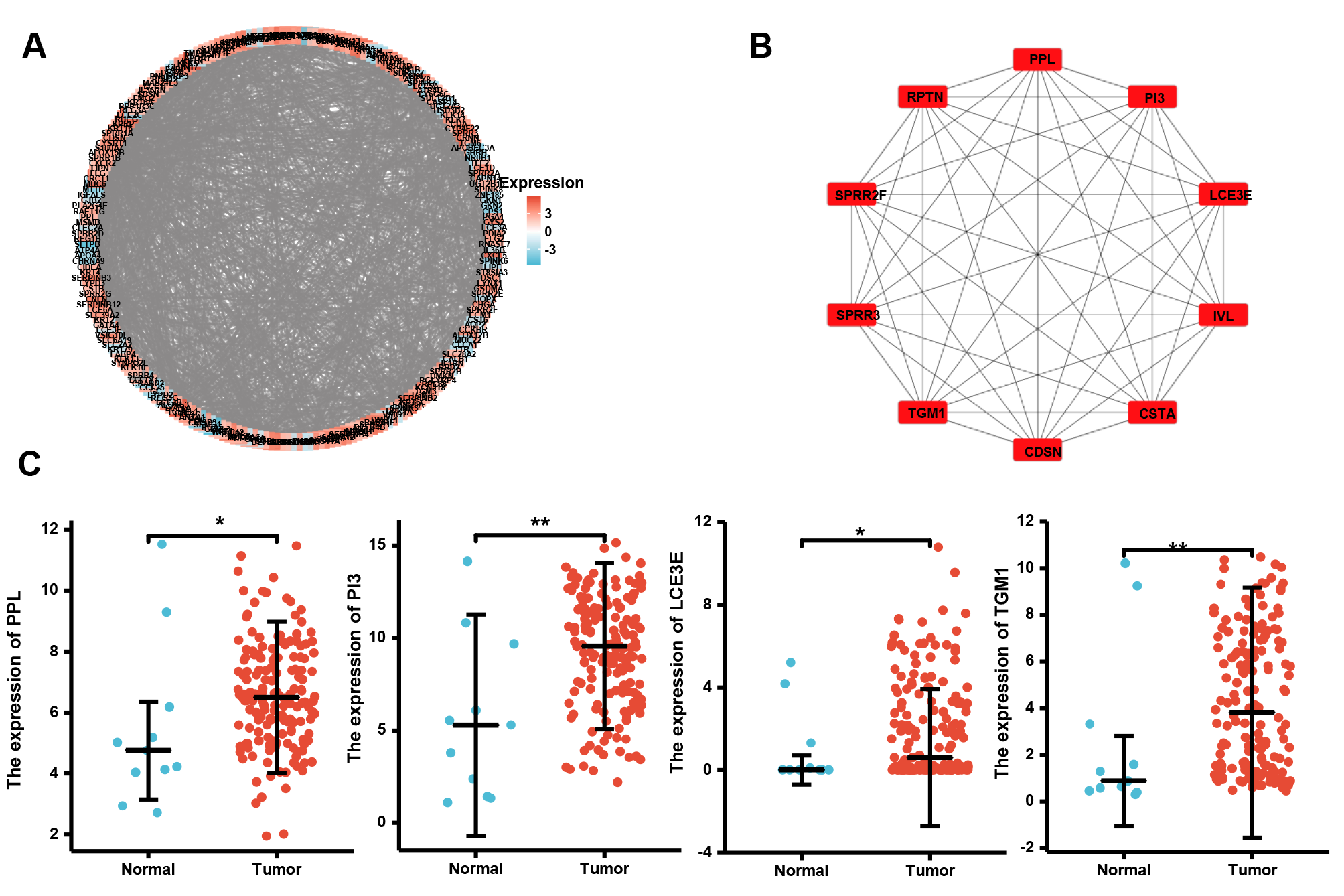

Supplement: Supplemental Material [file KCBT_A_2302162_SM7056.zip › Figure S2.tif]

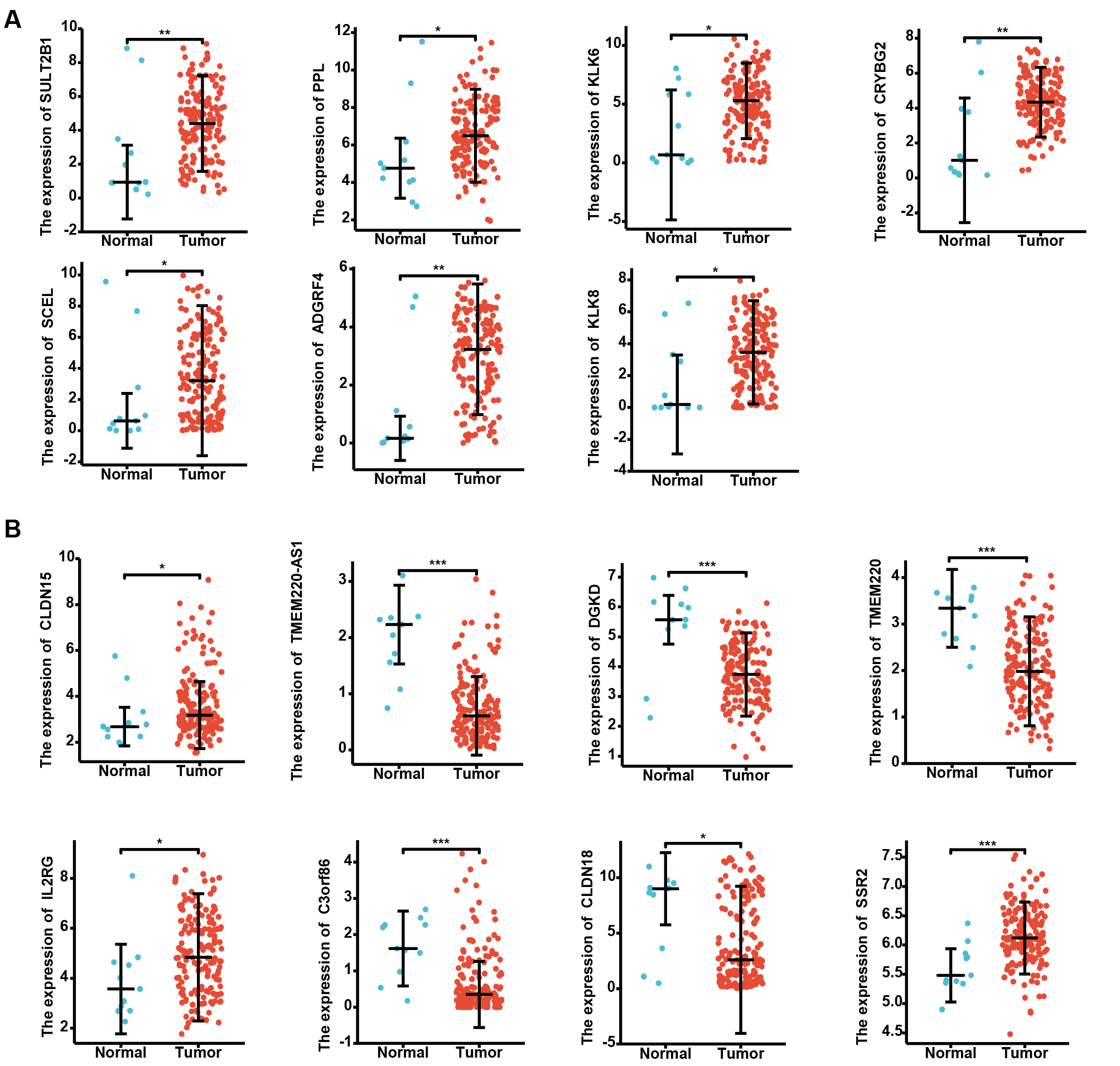

Supplement: Supplemental Material [file KCBT_A_2302162_SM7056.zip › Figure S3.tif]
